# Water-Based Automobile Paints Potentially Reduce the Exposure of Refinish Painters to Toxic Metals

**DOI:** 10.3390/ijerph15050899

**Published:** 2018-05-03

**Authors:** Der-Jen Hsu, Shun-Hui Chung, Jie-Feng Dong, Hui-Chung Shih, Hong-Bin Chang, Yeh-Chung Chien

**Affiliations:** 1 Department of Safety, Health and Environmental Engineering, National Kaohsiung University of Science and Technology, Kaohsiun 824, Taiwan; hsuderjen@nkfust.edu.tw; 2 Institute of Labor, Occupational Safety and Health, Ministry of Labor, New Taipei City 221, Taiwan; jongchung@mail.ilosh.gov.tw (S.-H.C.); B51208@yahoo.com.tw (H.-B.C.); 3 Graduate Master Program in Safety, Health and Environmental Engineering, Department of Safety, Health and Environmental Engineering, National Yunlin University of Science and Technology, Douliu 640, Taiwan; mike810528@gmail.com; 4 Department of Safety, Health and Environmental Engineering, Hungkuang University of Science and Technology, Shalu 433, Taiwan; hcshih0320@gmail.com; 5 Department of Safety, Health and Environmental Engineering, National Yunlin University of Science and Technology, Douliu 640, Taiwan

**Keywords:** paints, spray painting, toxic metals, lead, exposure assessment

## Abstract

Exposure to lead-containing dusts is a global public health concern. This work addresses an important issue of whether eco-friendly water-based paints reduce the exposure potential of auto-repainting workers to metals. With this aim, metal levels in automobile paints and worker metal exposure were measured using both solvent- and water-based paints. The levels of metals, and particularly Pb, Cr (total), Fe, and Cu, in solvent-based paints varied greatly among colors and brands. Lead concentrations ranged from below the detection limit (~0.25 μg/g) to 107,928 μg/g (dry film) across all samples. In water-based paints, the concentrations of Pb and Cr (total) were generally two to three orders of magnitude lower, but the concentrations of Al and Cu exceeded those in some solvent-based paints. The personal short-term exposure of workers who applied water-based paints of popular colors, such as black and white, were generally low, with Pb levels of less than <4 µg/m^3^ and Cr (total) levels of less than 1 µg/m^3^. Conversely, mean short-term exposure to Pb during the painting of a yellow cab using solvent-based paints were 2028 µg/m^3^, which was ~14 times the Taiwan short-term permissible exposure limit, while the mean level of exposure to Cr (total) was 290 µg/m^3^, which was well below the exposure limit. This study demonstrates that water-based paints reduce the exposure potential to lead, and highlights the importance of source control in limiting the toxic metals in paints.

## 1. Introduction

The fixing/replacing of automobile body parts typically requires re-painting, which is referred to as refinish painting. Refinishing differs from primary coatings that are used in car manufacturing with respect to its binders, solvents, and baking temperature [1,2]. Workers in auto-body repair shops are potentially exposed to various chemical, physical, ergonomic, sociological, and safety hazards [3,4,5]. Paint is a suspension of finely divided pigment particles in a liquid that is composed of a binder (resin) and a volatile solvent (or water), and normally contains additives that impart special characteristics [6]. Paint spraying causes aerosolization and subsequent inhalation exposure to these ingredients. Pigments that are commonly used in red paint, such as lead chromate molybdatesulphate red, and yellow paint, such as lead sulfochromate yellow, contain lead and chromate that have reproductive and carcinogenic effects. Although these pigments are listed as substances of very high concern (SVHC) by the European Chemicals Agency (ECHA) and have required authorization for use since May 2015, high-lead paints are still widely distributed around the world [7].

Employees in the fields of painting and paint manufacture have been found to have elevated risks of developing various cancers [8]. These findings are consistent with a report of the International Agency for Research on Cancer (IARC) that classified occupational exposure for painters as ‘carcinogenic to humans’, based mainly on epidemiological studies that identified increased risks of bladder and lung cancer [9,10,11]. However, such findings may be confounded by smoking and socioeconomic status, lack of exposure group effect, and publication bias [12].

Several investigations have assessed the exposure of workers in the metal and construction industries to heavy metals, mainly lead, during the scraping of old lead-based paints, spray painting, [13,14] and residential lead paint abatement projects [15,16,17,18]. However, studies of the exposure of automobile refinish painters to metal are few. One such study analyzed paint samples that were collected from an automobile repair shop at which a worker had been diagnosed with occupational lung cancer, and identified a few toxic chemicals, including chromate, lead, and SiO_2_. Environmental monitoring results have revealed air concentrations of 0.608 and 2.824 mg/m^3^ for Cr (total) and Pb, respectively [19]. However, relatively low exposures to Pb (0.05–5.75 μg/m^3^) and Cr (0.25–3.07 μg/m^3^) have also been identified for auto-body repainting workers [20]. Notably, occupational exposure to lead-related dusts can also be a public health concern through the take-home exposure pathway [21,22].

Water-based coatings have been used in the European Community (EC) since 1980–1990 to reduce paint consumption and limit volatile organic compounds (VOC) emissions. In other regions, their use is increasing [6,9]. The US and EC have set emission standards for VOC in automobile refinish coatings [23,24]. Thus, use of water-based paints not only potentially reduces worker exposure to toxic metals but VOC as well. However, studies regarding lead, chromate, and other toxic metal levels in such paints are scarce.

In Taiwan, nearly all small, privately-owned refinishing establishments utilize conventional solvent-based paint for both technical and economic reasons. Water-based paints are used only in the service departments of new car dealers (international brands). However, the outermost clear coat for water-based paint is nonetheless still solvent-based. The painting procedures differ slightly between applying solvent-based and water-based top coats. The application of water-based paint generally takes longer, which somewhat limits the use of water-based paint by small shops. The differences in paint ingredients and painting practices between the two paint types may affect worker exposure to metals.

This study aimed to assess the exposure potentials of automobile refinish workers, when using solvent-based or water-based paints, to toxic metals and examined airborne metal exposures during refinishing work.

## 2. Methods

### 2.1. Study Design

The potential exposure of workers to toxic metals was investigated through field surveys, worker interviews, and analyses of metal levels in paint samples, both solvent- and water-based. Actual worker paint color-specific exposures to metals were measured using personal and area samples during pre-treatment (sanding) and spray painting operations.

### 2.2. Worksite Surveys and Process Characterization

To better understand the repainting process, walk-through surveys and personnel interviews were first conducted on seven automobile repair establishments of various sizes in central and northern Taiwan, during June–September 2015. Five of the establishments (designated as stores S1–S5) that utilize solvent-based paint are small, privately owned shops, while the others (designated as stores W1 and W2) that utilized water-based paint are parts of the service department of new car dealers. Store S5 specializes in refinishing cabs (yellow color).

It was found that most of these establishments follow an 8-h work shift. The number of workers in small shops ranges from two to seven, while that in establishments associated with new car dealers is 10–20. Spraying booths, which provide filtered air and drying (baking) facilities, are used in all establishments. Small shops generally have only one booth for applying solvent-based paint, but shops that use water-based paints typically have two or three booths to meet high refinish demands and a slower turn-over rate that is associated with water-based paint application. The painting procedures differ slightly between applying solvent-based and water-based top coats. Repetitive spraying (~3 cycles) is common for applying solvent-based paint, whereas one to two cycles suffice to ensure coverage by water-based paint. The application time per painting cycle, to cover one entire medium-sized car, is 12–18 min. When solvent-based paint is used, an outer clear coat is applied shortly after the spraying of the last top coat, before baking, which is conducted at ~60 °C for 30 min. When water-based paint is used, a baking procedure (~60 °C for 20 min) follows the application of the top coat. After cooling to near room temperature, the clear coat is sprayed on, and the second baking is performed (~60 °C for 30 min). Typically, one or two experienced technicians are responsible for all painting tasks, but in some shops, each worker handles the complete refinish process. Accordingly, the potential exposure to metal dust varies significantly among shops. For example, in a shop that uses solvent-based paint and has a tight working schedule, and only one technician performs the spraying work, they may spend as long as 3–4 h in the spraying booth, which is the place with the highest exposure potential, if he sprays 3–4 loads/cars per day. In another shop, every technician might share the repainting work and so spray one load per day, so each individual worker has less exposure.

### 2.3. Collection and Analysis of Paint Samples

Paint samples were collected from the establishments during the visit. The colors of interest were red, yellow, blue, green, which have been shown to have high metal content potentials, and white, which was included mainly due to its popularity and for comparisons.

The sampled solvent-based paint samples, from the US, Germany, Italy, or Taiwan, were left over from recent jobs, and the dates of manufacture were unknown. All sampled water-based paints were from two German brands and obtained from the paint formulation rooms of the establishments. Their manufacturing dates were also unknown, although they were likely to have been made recently. Water-based paint samples were collected as single-color tinters and mixed with 50% (*w*/*w*) generic binder to mimic actual paints. All samples were collected in acid-washed glass vials with Teflon-liner cap and stored at room temperature until analysis.

Samples were pre-treated following the modified procedure from American Society for Testing and Materials to analyze the metal contents in paints [25]. Briefly, paint samples were applied thinly on a piece of glass plate (10 × 10 cm) and heated to 105 °C for 2 h in an oven to remove volatile solvents. Then, 1 g of the dry paint film was transferred to a crucible and heated slowly on a hotplate until the material was completely charred. Finally, the container was placed in a muffle furnace to form ash at 475 °C. For complete digestion, 5 mL of mixed acids (conc.HNO_3_/conc.HClO_4_:4/1 (*v*/*v*)) was added, and further additions of 2 mL during digestion as needed [26]. The digestion temperature was initially room temperature, which was held for 30 min, and increased to 150 °C for a few hours so that the solution would evaporate almost to complete dryness. The residue was transferred to a 25 mL flask with diluted acid for analysis. The metal analysis was performed using inductively coupled plasma optical emission spectrometry (ICP-OES) (Model Optima 5100 DV, PERKIN ELMER, Waltham, MA, USA). The recoveries of Pb, Cr, Cd, Al, Fe, Sr (strontium), and Cu following the above protocol were 103, 93, 95, 99, 106, 102, and 102%, respectively. The detection limits were 20.4, 10.1, 9.5, 12.9, 9.7, 16.3, and 15.4 μg/L for Pb, Cr, Cd, Al, Fe, Sr, and Cu, respectively. The lowest detectable level for calibration standards (in solution) is 0.005 mg/L, which corresponds to 0.25 μg/g (dry film) in samples.

### 2.4. Monitoring of Exposures of Worker to Metal

Paint color-specific personal exposures to metals were measured at five spray painting establishments (two used water-based and three used solvent-based paints) during September–October 2015. Two shops (S3 and S4) that had unstable working schedules were excluded from sampling. Typical working schedule was followed during sampling, thus exposure potentials under normal shift was assessed for each shop.

Personal samples were taken during sanding and spray painting while area samples were taken inside the painting booth during spraying, at a height of ~150 cm. The sampling time for personal monitoring was normally 12–18 min, a period which covered one spraying cycle for an entire car or extensive loading of auto parts. However, multiple samples were usually taken because 2–3 spraying cycles are required to complete the painting process. The sampling time for area measurement was the duration of an entire spraying task, typically 20–60 min. Thus, a total of 28 personal and 12 area samples were taken and analyzed.

The monitoring generally followed the reference method for collecting total airborne particulates [27]. Paint particles were collected on mixed cellulose ester filter paper (0.8 μm pore size) in a 37 mm cassette (SKC, Covington, GA, USA) that was connected to a sampling pump that ran at ~4 L/min to ensure sufficient dust load for analysis. The sample filters were firstly weighed after desiccation to assess the dust loading, and then the metal analysis of the paints was carried out, as described earlier. The lowest quantifiable level of the metal in air was 2.5 µg/m^3^, based on the lowest detectable level for the analytical protocol and a typical sampling volume of 60 L (=15 min × 4 L/min). The metal levels in reagent blanks and blank filters were all below the detection limits.

### 2.5. Data Analysis

The metal concentrations in the paint samples were expressed as μg/g of dry paint film, or µg/m^3^ for the metal concentrations in the airborne paint particles. Worker tasked-based exposure concentrations were calculated based on actual sampling period, i.e., from 10 to 64 min, that covered the periods for specific work tasks. These levels were then normalized to 15-min as short-term exposures that can be compared with short-term exposure limits (STEL). Data was presented as minimum, maximum, median, mean, and standard deviation values. Half of the detection limit value was used to calculate mean if sample concentrations were below the method detection limit. Statistical differences between groups were tested using a nonparametric Mann–Whitney *U* test. The *α* was set at 0.05 level, but Holm’s sequential Bonferroni correction for significance level was applied for multiple comparisons to avoid over-claiming of statistical significance. Workplace STELs are determined based on the Permissible Exposure Limit—Time-Weighted Average (PEL-TWA) and excursion factors set by Taiwan Occupational Safety and Health Administration. The value for Iron is set based on iron oxide. The PEL-TWA for Sr is not available.

## 3. Results and Discussion

### 3.1. Metal Levels in Car Refinishing Paints

The metal concentrations, along with other information such as brand, country of origin, and collection site, in selected solvent-based and water-based paints are listed in the Supplement Tables S1 and S2.

Of the samples of solvent-based paints (*n* = 23), 91.3, 65.2, 91.3, 100, 56.5, and 0% contained Pb, Cr (total), Al, Fe, Cu, and Cd, respectively, and the median (mean) concentrations were 159 (12,557), 1210 (2618), 1674 (2065), 191 (2560), and 4786 (5082) μg/g (dry film), respectively. The levels of metals, particularly on Pb, Cr (total), Fe, and Cu, varied greatly among colors and brands. For example, lead concentrations ranged from below the detection limit (~0.25 μg/g) to 107,928 μg/g. The cadmium levels in all samples were lower than the detection limit, and are thus not discussed further. Two domestic brands (E-YUAN and Butterfly) of commercially available yellow paints had high lead levels (>17,000 μg/g). Lead concentrations in paints were closely correlated (*r* = 0.94) with those of Cr (total), indicating that lead(II) chromate (pigment “chrome yellow”) may be present in many samples. There were 11 samples (47.8%) having a lead level above 90 ppm, a limit value set by many countries including the US.

Similarly, 85.0, 100, 100, 100, 90.0, 0, and 85.0% of the water-based paint samples (*n* = 20) contained Pb, Cr (total), Al, Fe, Cu, and Cd, respectively, with the median (mean) concentrations being 2.08 (7.53), 3.39 (4.38), 21.2 (855.2), 60.0 (839.9), 1.57 (7441), and 1.94 (74.1) μg/g (dry film), respectively. Overall, the concentrations of Pb and Cr (total) in water-based paints were low, in the range of a few to low tenths of μg/g, and the variations were smaller than those in solvent-based paints. However, the concentrations of Al and Cu may exceed those in solvent-based paints.

Table 1 compares metal concentrations in car refinishing paints of various colors. Analytical results for solvent-based paint samples reveal that yellow paints had the highest Pb, Cr (total), and Fe concentrations, with mean levels of 29,108, 5563, and 7598 μg/g (dry film), respectively; these were followed by red paints, with mean levels of Pb and Cr (total) of 14,353 and 890 μg/g, respectively. The white and blue paints had the highest mean concentrations of Al (5458 μg/g) and Cu (9741 μg/g), respectively. Green paints also had relatively high levels of Cu, averaging at 5406 μg/g. However, statistically significant differences in concentration among colors for solvent-based paints were only found for Cu between yellow and green (*p* = 0.004), and between yellow and blue (*p* = 0.006) colors, likely due to large variations in metal concentration and small sample size in each color group. Large variations in metal concentration in some color groups such as yellow and red reflect the fact that low-Pb pigment may have been used by major paint manufacturers.

Chromate, lead, and Sr have been found in paint samples (solvent-based) that were collected from a shop that had had a case of occupational lung cancer [19]. Lead, Cr(6+) (total Cr), and Sr levels in orange-yellow, yellow, and bright yellow paints have been to be 173,568, 78,105, and 186,437 ppm; 18,295 (43,416), 14,085 (14,085), and 10,935 (36,302) ppm; and 46, 141, and 267 ppm, respectively. However, only trace levels have been detected in eco-friendly paints [19]. The results of this investigation are consistent with these findings.

The blue water-based paints had the highest concentrations of Pb and Cu, with means of 29.0 and 36,202 μg/g, respectively, while the yellow paints had the highest mean Fe concentration of 2545 μg/g. Black and white paints had the highest mean total concentrations of Cr (13.8 μg/g) and Al (7861 μg/g), respectively. No statistically significant differences in concentration among colors across metals could be identified for water-based paints.

Although water-based paints claim to contain little or no Pb and chromate, literature reports on their concentrations are scarce. Current data indicate that the levels of metals, particularly Pb and Cr (total), in water-based paints are generally two to three orders of magnitude lower than those found in solvent-based paints, and especially red and yellow paints. Yellow water-based paints also had lower mean Pb concentration, but not reaching statistical significance level. Similarly, green water-based paints had significant lower Pb (*p* = 0.025) concentration, and blue paints had significant lower Al (*p* = 0.034) and Fe (*p* = 0.032) concentrations than their solvent-based counterparts. Conversely, the concentration of certain metals in water-based paints exceeded that in solvent-based paints. For example, blue paints (water-based) had significant higher Cu (*p* = 0.034) and total Cr (*p* = 0.019) concentrations than those of solvent-based blue paints, even though the total Cr concentrations in both paint types were low.

A study comparing the metal concentrations of paint dusts in paint manufacturers found significantly higher Pb concentrations (15,680 ± 11,780 μg/g vs. 57.46 ± 22.42 μg/g) in solvent-based paints manufacturers than in water-based paint plants [28]. This finding is consistent with current results that water-based paints have lower lead levels.

Collectively, these findings suggest that the type of paint (water- or solvent-based), in addition to its brand and color, may provide guidance on the exposure potential of refinishing painters to metals in this population.

### 3.2. Exposure of Workers to Metal Dusts

In this investigation, the exposures of workers to metals were measured for specific paint colors, including the commonly used colors of white, silver, and black. The summary results of exposure monitoring (Table 2 and Table 3) showed that the personal tasked-based (sampling time from 9 to 66 min) exposures to heavy metals of workers who apply water-based paints of common colors were generally low, with Pb levels of under 3 µg/m^3^ and Cr (total) levels of less than 1 µg/m^3^. The exposure levels to Fe and Al were slightly higher. Exposures to Pb and Cr (total) in solvent-based paints were also low, being generally less than a few µg/m^3^ for workers who are spraying paints of popular colors such as black and white. Such exposure levels are similar to those in literature data, that is, 0.05–5.75 μg/m^3^ for Pb, and 0.25–3.07 μg/m^3^ for total Cr [20]. However, the exposures to Fe and Al in white paint were higher, with median levels of 29.87 and 105.0 µg/m^3^, respectively. This finding is consistent with previous results that revealed only Fe and Al in grey paint in a repainting shop [19].

The mean tasked-based exposures to Pb during the painting of a cab using solvent-based yellow paint (top coat) was 2036 µg/m^3^, which corresponds to a 15-min short-term exposure of 2028 µg/m^3^ (Table 4). This level was ~14 times the Taiwan short-term permissible exposure limit of 150 µg/m^3^. This high short-term exposure will result in an 8-h TWA exposure of 190 µg/m^3^ (assuming zero exposure during non-spraying period), which also exceeds the PEL-TWA of 50 µg/m^3^. During this same sampling period, the mean short-term exposure level of Cr (total) was 290 µg/m^3^, which was well below the exposure limit. These exposure levels were similar to those in the literature, in which levels in air (obtained through area sampling inside a spraying booth) of 608 and 2824 µg/m^3^ for Cr (total) and Pb, respectively, were measured during the spraying of yellow paint [19]. The exposures of spraying workers to metals seem to be related to the levels of the metals in the paints being used. For example, the lead level in the paint that was used during personal exposure sampling for a subject was 17,134 µg/g (“Cab yellow” paint from brand E-YUAN, Table S1). This top coat yielded high personal and area (inside the spraying booth) levels of 2345–3593 µg/m^3^ and 1682 µg/m^3^, respectively (Table S3). Similarly, a Pb concentration of 2824 µg/m^3^ inside booth (area sampling) has also been found to arise from spraying yellow paint (Pb concentrations of 78,105–186,437 ppm) [19].

For exposure during sanding, comparisons were only made between the stores that use different paint types, as the types of paint being sanded were not confirmed. The analysis results indicated that the exposure to Cr (total) in the two stores that handle solvent-based paint was significantly higher (*p* = 0.044) than those using water-based paint.

Store-wise comparisons of short-term exposure levels during painting revealed that the Pb and Cr (total) exposures in a store (S5) that specializes in repainting yellow cab were higher than other two stores (S1 and S2) that also apply solvent-based paints. However, such differences were only statistically significant between stores S1 and S5, with *p* value of 0.013 and 0.014 for Pb and Cr (total), respectively. For the two stores that utilize water-based paints, exposures were only significantly different for Sr (*p* = 0.047) and Cu (*p* = 0.014). Likewise, multiple comparisons across all stores indicated that the exposure to Cu in store W1 (using water-based paint) was significantly higher (*p* = 0.007) than that in store S1 (using solvent-based paint).

Blue water-based paints usually have high levels of Cu (mean = 36,202 µg/g; maximum = 56,123 µg/g, Table 1). Based on the relationship between the metal levels in paint and worker personal exposures identified above, such a high Cu level in blue paint may result in worker exposure levels of thousands of µg/m^3^ during spraying, likely exceeding the Taiwan short-term exposure limit of 2000 µg/m^3^ for Cu.

Additionally, the 15-min short-term exposures to total particulate/dust during sanding were below the Taiwan STEL of 15,000 µg/m^3^ for total dust. Sanding of a new, unpainted bumper resulted in exposure to particles that exceeded half of the permissible exposure limit for dust (total), and such dust contained a small amount of Pb. The short-term exposures to total paint particles were greatest during spraying, with eight (8/20 = 40%) personal exposure measurements exceeding the short-term exposure limit, mostly during spraying solvent-based paint (Table 4).

Paint is applied in a ventilated spraying booth using a pressurized spray gun. The pressure is set mostly based on the worker’s experience. The US OSHA has recommended the use of a high-volume, low-pressure (HVLP) spray painting gun and a fully downdraft booth with a downward air flow of ~0.4 m/s, to reduce the concentrations of paint overspray. Although not measured in this study, the (downward) air flow speed of 0.30–0.35 m/s that is used by most facility manufacturers seems insufficient to remove paint aerosols that are generated during spraying—particularly in those shops that had high metal exposure levels. Personal protective equipment (PPE), such as dust masks, is normally used to control exposure to paint particles. However, the PPE management programs and thus the effectiveness of practices vary significantly among shops. At private small shops, procedures for the selection, fitness testing, and replacement of PPE are lacking, likely leading to insufficient control. Additionally, as protective clothing and gloves are hardly worn in these small shops, contamination of body parts and clothing with paint residues is likely. This may cause worker dermal exposure and can subsequently pass on to other locations/persons, causing involuntary exposures.

### 3.3. Regulatory Considerations

No legal standard for the lead (and other metals) contents in consumer or industrial paints exists in Taiwan. However voluntary “Green-Mark” criteria set by the Taiwan EPA state that Hg, Pb, Cd, Cr(6+), and As concentrations should not exceed 2, 2, 2, 3, and 3 ppm, respectively, in oil-based paints, and should be non-detectable (<2 ppm) in water-based paints. Governments rarely set standards for metal levels in automobile paints. One such regulation from China requires that Pb, Cr(6+), Hg, and Cd levels do not exceed 1000, 1000, 1000, and 100 mg/kg, respectively, in either water- or solvent-based paints.

Notably, the lead levels in household decorative paint samples that were collected in Brazil before lead paint was regulated were found to be high as 170,000 ppm, falling to below the detection level (<9 ppm) after the regulation for residential paint came into force [29,30]. This action emphasizes the importance of regulatory intervention to reduce the potential exposure of users.

Global programs, such as the WHO/UNEP Global Alliance to Eliminate Lead Paint initiative, have been catalyzing efforts to achieve international goals to minimize occupational exposure to lead paint. Unfortunately, many countries including Taiwan are not parts of the program, nor do these countries set stringent regulations on lead-containing paints.

## 4. Conclusions

Exposure to lead-containing dusts in the workplace is not only an occupational hazard, but can also be a public health concern owing to potential take-home exposure pathway, and should thus be minimized. Water-based automobile coating is mainly used for its low VOC-consuming property, however, it can also limit exposure to lead and other toxic ingredients if being properly formulated.

The current measurements demonstrate that the levels of toxic metals, particularly Pb, in some solvent-based paints can be extremely hazardous, while those in water-based paints are generally two to three orders of magnitude lower than in solvent-based paints, especially red and yellow paints. Workers who use solvent-based yellow-colored automotive paints are at high risk, particularly due to lead exposure. Collectively, these findings suggest that water-based paints reduce the exposure potentials to lead and other toxic metals, except copper, and highlight the need for source control to limit toxic metals in automobile-refinish paints.

Therefore, this study calls for the immediate control of occupational metal-related hazards, particularly in small auto-repairing shops. Low-metal automobile paints are available on the market and should be adopted. Nonetheless, before such a goal is realized, effective PPE may be the best means of hazard control for painters. Biological monitoring of the body burden of metals and screening for high-risk workers through a medical surveillance program are also suggested to control related health risks.

## Figures and Tables

**Table 1:** Metal concentrations in car refinishing paints grouped by colors.

Paint Color *	*n*	Metal Concentration (μg/g Dry Film) **					
		Pb	Cr (Total)	Al	Fe	Cu	Sr
Solvent-based							
(1) Red	5	6.34~47,821 **	24.5~2272	ND~331	32.6~3156	ND~3.1	NA
		(65.9)	(239)	(179)	(463)	(ND)	
(2) Yellow	6	ND~107,928	ND~22,152	ND~5346	139~38,503	ND~44.7	NA
		(12,411)	(1756)	(2842)	(2068)	(ND)	
(3) Blue	4	32.9~159	ND~ND	271~2620	145~1448	7336~13,977	NA
		(41.4)	(ND)	(1409)	(168)	(8826)	
(4) Green	5	27.4~16,160	ND~1210	569~2516	99~4450	2753~9662	NA
		(54.9)	(6.2)	(1648)	(148)	(4786)	
(5) White	3	ND~210	ND~105	4413~6097	162~266	ND~17.1	NA
		(38.1)	(15.4)	(5863)	(222)	(ND)	
Significance ***		-	-	-	-	(3) > (2) (4) > (2)	-
Water-based							
(A) Red	4	ND~0.31	0.3~6.42	0.26~75.0	21.8~182	0.16~4.82	0.32~6.77
		(0.26)	(0.78)	(4.03)	(74.5)	(0.86)	(3.66)
(B) Yellow	6	0.3~11.5	0.79~10.0	2.09~73.8	50.5~14,833	ND~0.44	0.36~878
		(2.16)	(6.08)	(24.0)	(76.2)	(0.24)	(1.74)
(C) Blue	3	18.7~34.2	0.37~4.42	0.34~9.64	9.59~122	24,653~56,123	ND~1.17
		(34.1)	(3.18)	(5.97)	(58.8)	(27,830)	(ND)
(D) Green	3	2.08~13.4	0.82~4.27	46.3~578	23.2~455	854.1~12,383	1.94~178
		(12.2)	(3.6)	(455)	(217)	(12,089)	(174)
(E) White	2	ND	0.55~1.05	7386~8335	24.1~25.4	ND~0.03	ND~0.9
			(0.8)	(7861)	(24.7)	(0.02)	(0.45)
(F) Black	2	0.27~0.33	6.27~21.3	3~6.58	56.3~183	1.71~4.62	0.79~3.45
		(0.3)	(13.8)	(4.79)	(120)	(3.17)	(2.12)
Significance ***		(1) > (A)	(1) > (A)	(3) > (C)	(3) > (C)	(C) > (3)	-
		(4) > (D)	(C) > (3)				

* Color-grouping is subjective to the researcher. Orange is categorized as red, ochre as yellow; ** Minimum~Maximum (Median), ND: below method detection limit, NA: not measured. In case of ND, half of the detection limit value is used to calculate mean; *** Statistical comparisons between groups using Mann–Whitney *U* test. The *α* level is 0.05, but Holm’s sequential Bonferroni correction for significance level is applied for multiple comparisons.

**Table 2:** Worker task-based airborne exposures to metals in automotive re-painting using water-based paints.

Activity/Type *	Paint Color	Sampling Time (min)	*n*	Air Concentration (μg/m^3^) **						
				Mass (Total)	Pb	Cr (Total)	Fe	Sr	Cu	Al
(1) Sanding, P	Various colors	15~16	5	1741~9320	ND~3.55	ND~0.09	ND~76.43	ND~2.18	ND~2.07	ND~101.1
				(3202)	(1.71)	(ND)	(21.55)	(ND)	(ND)	(16.85)
				(4005–3048)	(1.46–1.5)	(0.03–0.04)	(30.3–33.8)	(0.44–0.97)	(0.42–0.92)	(36.9–45.1)
(2) Spraying, P	White	16~18	2	9671~19,872	1.1~1.46	ND	23.1~69.2	ND~2.75	ND~0.11	ND~74
				(14,772)	(1.28)		(46.2)	(1.38)	(0.06)	(37)
				(14,772–7213)	(1.28–0.25)		(46.2–32.6)	(1.38–1.94)	(0.06–0.08]	(37–52.3)
(3) Spraying, P	Black metallic	16~20	2	4253~13,445	ND	ND	ND	ND~2.74	1.2~9.98	0.8~8.53
				(8849)				(1.37)	(5.59)	(4.67)
				(8849–6500)				(1.37–1.94)	(5.59–6.21)	(4.67–5.47)
(4) Spraying, P	Black	12~15	2	3537~5992	0.5~0.56	ND~0.86	9.22~29.6	ND	ND	ND~26.6
				(4765)	(0.53)	(0.43)	(19.4)			(13.3)
				(4765–1736)	(0.53–0.04)	(0.43–0.61)	(19.4–14.4)			(13.3–18.78)
(5) Spraying, P	Various colors	16~17	2	6608~12,352	ND~1.78	ND~0.05	27.4~74.3	ND~2.06	ND~0.08	130.1~309.2
				(9480)	(0.89)	(0.03)	(50.9)	(1.03)	(0.04)	(220)
				(9480–4062)	(0.89–1.26)	(0.03–0.03)	(50.9–33.2)	(1.03–1.45)	(0.04–0.05)	(220–127)
(6) Spraying, A	Various colors	13~19	6	1582~14,609	ND~2.48	ND~0.41	ND~28.3	ND	ND~10.3	ND~74.6
				(2295)	(1.08)	(ND)	(4.6)		(ND)	(20.1)
				(5880–6111)	(1.03–0.97)	(0.07–0.17)	(10.4–13)		(2.13–4.13)	(23.7–27.5)
(7) Spraying, P	All colors (Store W1)	16~20	4	4253~19,872	ND~1.1	ND	ND~69.2	ND~2.75	0.08~9.98	0.8~309
				(12,899)	(ND)		(13.7)	(2.4)	(0.66)	(41.2)
				(12,481–6410)	(0.28–0.55)		(24.2–32.7]	(1.89–1.3)	(2.84–4.79)	(98.1–145)
(8) Spraying, P	All colors (Store W2)	12~17	4	3537~9671	0.5~1.78	ND~0.86	9.22~74.3	ND	ND	ND~130
				(6300)	(1.01)	(0.03)	(26.4)			(13.3)
				(6452–2523)	(1.08–0.64)	(0.23–0.42)	(34.1–28.2)			(39.2–61.9)
Significance ***				-	-	-	-	(7) > (8)	(7) > (8)	-

* Sanding, P: personal sampling of scraping old paint/sanding of filling/sanding of new, un-painted bumper; Spraying, P: spray painting, personal sampling; Spraying, A: spray painting, areal sampling inside booth; ** Minimum~Maximum (Median) (Mean-SD), ND: below method detection limit, NA: not measured. Worker tasked-based exposure concentrations were calculated based on actual sampling period. In case of ND, half of the detection limit value is used to calculate mean; *** Statistical comparisons between groups using Mann–Whitney *U* test. The *α* level is 0.05, but Holm’s sequential Bonferroni correction for significance level is applied for multiple comparisons.

**Table 3:** Worker task-based airborne exposures to metals in automotive re-painting using solvent-based paints.

Activity/Type *	Paint Color	Sampling Time (min)	*n*	Air Concentration (μg/m^3^) **						
				Mass (Total)	Pb	Cr (Total)	Fe	Sr	Cu	Al
(A) Sanding, P	Black/Mustard	9~16	2	1569~1830 (1700) (1700–185)	ND	1.36~2.42 (1.89) (1.89–0.75)	28.5~41 (34.7) (34.7–8.8)	ND	ND	2.7~2.83 (2.77) (2.77–0.09)
(B) Spraying, P	White	16~19	4	1321~32,189 (22,588) (19,671–13,071)	ND	ND~1.49 (0.61) (0.68–0.66)	24.7~41.1 (29.9) (31.4–7.38)	ND	ND	5.5~140 (1059) (88.8–57.9)
(C) Spraying, P	Black	28~57	2	4255~16,139 (10,197) (10,197–8403)	ND~0.56 (0.28) (0.28–0.39)	ND~0.28 (0.14) (0.14–0.2)	0.47~32.1 (16.3) (16.3–22.4)	ND	ND	0.59~4.59 (2.59) (2.59–2.83)
(D) Spraying, P	Yellow	13~15	3	6346~20,000 (11,167) (12,504–6925)	170~3593 (2345) (2036–1732)	19.1~604 (250) (291–294)	8.38~15.9 (11.6) (11.9–3.76)	ND	ND	25.9~43.6 (26.9) (32.1–9.93)
(E) Spraying, P	Various colors	10~16	3	2511~29,532 (4000) (12,014–15,189)	ND~39.1 (4.17) (14.4–21.5)	0.16~3.52 (1.77) (1.82–1.68)	15.97~42.55 (21) (26.5–14.1)	ND	ND	62.6~208 (161) (143.9–74.3)
(F) Spraying, A	Various colors	16~64	6	472~10,441 (3637) (4425–3864)	ND~267 (0.27) (44.7–109)	ND~43.3 (ND) (7.24–17.7)	ND~21.9 (1.81) (6.98–9.25)	ND	ND	0.99~31.3 (3.08) (8.15–11.8)
(G) Spraying, P	All colors (Store S1)	16-57	5	4255~32,189 (23,612) (22,230–10,931)	ND~4.17 (ND) (0.95–1.82)	ND~1.77 (0.91) (0.90–0.75)	0.47~42.6 (32.9) (28.8–17.1)	ND	ND	0.59~161 (105) (102–61.6)
(H) Spraying, P	All colors (Store S2)	14~28	3	1321~16,139 (2511) (6657–8233)	ND	ND~0.28 (0.16) (0.15–0.14)	16~32.1 (24.7) (24.2–8.08)	ND	ND	4.59~208 (5.5) (72.8–117)
(I) Spraying, P	All colors (Store S5)	10~15	4	4000~20,000 (8757) (10,378–7074)	39.1~3593 (1258) (1537–1731)	3.52~604 (134) (219–280)	8.38~21 (13.7) (14.2–5.46)	ND	ND	25.9~62.6 (35.2) (39.7–17.3)
Significance ***				-	(I) > (G)	(I) > (G)	-	-	-	-
						(A) > (1) ****			(7) > (G) ****	

* Sanding, P: personal sampling of scraping old paint/sanding of filling/sanding of new, un-painted bumper; Spraying, P: spray painting, personal sampling; Spraying, A: spray painting, areal sampling inside booth; ** Minimum~Maximum (Median) (Mean-SD), ND: below method detection limit, NA: not measured. Worker tasked-based exposure concentrations were calculated based on actual sampling period. In case of ND, half of the detection limit value is used to calculate mean; *** Statistical comparisons between groups using Mann–Whitney *U* test. The *α* level is 0.05, but Holm’s sequential Bonferroni correction for significance level is applied for multiple comparisons; **** (1) represents data of sanding, personal sampling, and (7) represents data of spray painting, personal sampling on Store S5 (Table 2).

**Table 4:** Worker short-term exposures to metal particulates during car-refinish painting.

Sample Type *	Paint Color	Sampling Time (min)	Worker **	Short-Term Exposure Concentration (μg/m^3^) ***						
				Mass (Total)	Pb	Cr (Total)	Fe	Sr	Cu	Al
Short-Term Exposure Limits (STEL) ***				15,000	150	2000	15,000	NA	2000	22,500
Water-based paint										
S1	Blue, grey	15	W1-1	1741	ND	ND	ND	ND	ND	ND
S1	Blue, grey	15	W1-1	3202	ND	ND	ND	ND	ND	ND
P1	Black, metallic	16	W1-2	14,341	ND	ND	ND	ND	10.65	9.10
P1	Black, metallic	20	W1-2	5671	ND	ND	ND	3.65	1.60	1.07
P2	Black, metallic	18	W1-2	17,531	ND	ND	ND	ND	12.37	ND
P1	White	18	W1-3	23,846	1.32	ND	83.0	3.30	0.13	88.74
P1	Silver	16	W1-3	13,175	ND	ND	29.33	2.20	0.90	329.8
P2	White	19	W1-3	2004	ND	ND	10.46	ND	ND	20.00
S3	Black	15	W2-1	9320	2.03	ND	53.57	ND	ND	66.54
S3	Black	15	W2-2	2329	1.71	0.07	76.43	ND	ND	101.1
S2	Silver	16	W2-3	3661	3.79	0.10	22.99	2.33	2.21	17.97
P1	Black	15	W2-3	5992	0.50	0.86	29.61	ND	ND	ND
P2	Black	15	W2-3	2663	1.53	0.41	0.93	ND	2.44	ND
P1	White, pearl	16	W2-3	10,316	1.56	ND	24.64	ND	ND	ND
P2	White, pearl	16	W2-3	13,689	2.65	ND	30.22	ND	ND	79.57
P1	Beige+ClearCoat	17	W2-3	7489	2.02	0.06	84.25	ND	ND	147.5
P2	Beige+ClearCoat	18	W2-3	2003	0.92	ND	29.77	ND	ND	32.60
P1	Black	12	W2-3	2830	0.45	ND	7.38	ND	ND	21.25
P2	Black	13	W2-3	1669	1.20	ND	ND	ND	ND	21.16
Solvent-based paint										
P1	Black	57	S1-1	16,169	2.13	ND	1.79	ND	ND	2.24
P2	Black	57	S1-1	25,882	2.05	ND	8.66	ND	ND	3.76
P2	White	66	S1-1	6829	2.38	ND	5.85	ND	ND	41.36
P1	White	18	S1-1	28,334	ND	0.37	39.53	ND	ND	125.6
P1	White	16	S1-1	23,001	ND	0.97	28.59	ND	ND	112.2
P1	White	16	S1-1	34,335	ND	1.59	43.85	ND	ND	149.0
P1	White+ClearCoat	16	S1-1	31,501	4.45	1.89	45.39	ND	ND	171.5
S2	Black	9	S2-1	1098	ND	0.82	24.58	ND	ND	1.62
P2	Silver	16	S2-1	503.1	ND	ND	ND	ND	ND	1.08
P1	Silver	14	S2-1	2344	ND	0.15	14.91	ND	ND	194.4
P1	White	19	S2-2	1673	ND	ND	31.22	ND	ND	6.97
P2	White	20	S2-2	13,921	ND	ND	29.13	ND	ND	41.76
S1	Mustard	16	S2-3	1674	ND	2.58	30.42	ND	ND	3.02
P1	Black	28	S2-3	30,126	ND	0.52	59.94	ND	ND	8.57
P2	Black	28	S2-3	10,437	ND	0.17	28.60	ND	ND	5.47
P2	Yellow	64	S5-1	7177	1140	184.9	4.74	ND	ND	13.78
P1	(Sealant)	10	S5-1	2667	26.0	2.35	13.99	ND	ND	41.71
P1	Yellow, light	13	S5-1	5500	147.5	16.57	13.76	ND	ND	23.30
P1	Yellow	15	S5-1	11,167	2345	249.5	11.57	ND	ND	43.56
P1	Yellow	15	S5-1	20,000	3593	603.6	8.38	ND	ND	25.88

* S1: scraping old paint, S2: sanding of filling, S3: sanding of new, un-painted bumper, P1: spray painting, personal sampling, P2: spray painting, area sampling inside booth; ** Shop-individual worker. ND: below method detection limit; *** Adjusted to 15-min, short-term exposure for comparisons with STEL.

## Supplementary Materials

The following are available online at http://www.mdpi.com/1660-4601/15/5/899/s1, Table S1: Metal concentrations in selected solvent-based automobile refinishing paints; Table S2: Metal concentrations in selected water-based automobile refinishing paints; Table S3: Worker tasked-based airborne exposures to metal particles during car refinish painting.
