# Supplementary material for: Water-Based Automobile Paints Potentially Reduce the Exposure of Refinish Painters to Toxic Metals
Source: Int J Environ Res Public Health. 2018 May 3;15(5):899. doi: 10.3390/ijerph15050899 (PMC5981938; doi:10.3390/ijerph15050899)
Supplement: Supplementary file 1 [file ijerph-15-00899-s001.pdf]

Supplement Materials

Table S1. Metal concentrations in selected solvent-based automobile refinishing paints

Color*	Brand	C.O.**	Shop***	Metal concentration (µg/g dry film) Pb	Cr(total)	Al	Fe	Cu	Cd
Orange red	DeBeer	US	S1	47821	1886	179	63.6	ND	ND
Bright red	DeBeer	US	S1	30.5	239	167	32.6	ND	ND
Yellow	DeBeer	US	S1	107928	22152	2925	171	ND	ND
Deep blue	MaxMeyer	IT	S1	44.7	ND	271	145	13977	ND
Deep green	MaxMeyer	IT	S1	29.7	ND	569	148	6372	ND
Yellow	MaxMeyer	IT	S2	12411	2035	2153	38503	ND	ND
Dark red	MaxMeyer	IT	S2	65.9	24.5	225	2009	ND	ND
Light green	Standox	DE	S2	16160	1210	1709	191	2753	ND
White	Glasurit	DE	S3	210	105	4413	266	17.1	ND
Opaque yellow	Glasurit	DE	S3	ND	ND	6.30	3437	ND	ND
Bright red	Glasurit	DE	S3	23842	2272	331	3156	3.10	ND
Dark green	MaxMeyer	IT	S3	27.4	ND	674	4450	3455	ND
Light yellow	E-YUAN	TW	S3	7616	1756	5346	2068	ND	ND
Bluish green	E-YUAN	TW	S3	489	112	2516	107	4786	ND
White	UNO-HD	DE	S3	38.1	15.4	6097	222	ND	ND
Indigo blue	UNO-HD	DE	S3	38.1	ND	2620	168	8881	ND
Pearl blue	Centari	DE	S4	159	ND	1143	168	7336	ND
Pearl white	Centari	DE	S4	ND	ND	5863	162	ND	ND
Sapphire blue	Centari	DE	S4	32.9	ND	1674	1448	8771	ND
Dark green	Centari	DE	S4	54.9	6.20	1648	99	9662	ND
Cab yellow	E-YUAN	TW	S5	17134	1493	2842	139	ND	ND
Toyota yellow	Butterfly	TW	-	29559	5940	ND	1268	44.7	ND
Sunrise red	Butterfly	TW	-	6.34	28.83	ND	463	2.49	ND

*Colors are subjective to the researcher, except for Cab Yellow, Toyota Yellow and Sunrise Red, which are translated according to the product labels (commercially-available paints); **Country of origin. US: USA; DE: Germany; IT: Italy; TW: Taiwan; ***Samples were obtained from five privately-owned repair shops, and were left-over from previous uses. Shop S5 specializes in taxi repainting; - obtained from resale store. ND: below method detection limit.

Table S2. Metal concentrations in selected water-based automobile refinishing paints

Color (Tinter)[*]	Brand[**]	Color code	Metal concentration (μg/g dry film)						
			Pb	Cr(total)	Al	Fe	Cu	Cd	Sr
Yellow	C	WB42	2.91	7.11	73.8	91.1	0.01	ND	2.01
Opaque yellow	C	WB41	5.52	10	2.09	181	ND	ND	1.5
Orange yellow	C	WB46	0.3	5.04	20	61.2	0.44	ND	0.36
Bright red	C	WB60[***]	ND	8.74	2.55	118	1.08	ND	0.60
Bright red	C	WB60	0.23	6.42	1.49	36	0.29	ND	0.32
White H/S	C	WB01	ND	1.05	7386	24.1	0.03	ND	ND
Green Shade Blue	C	WB27	24.1	3.18	5.97	58.8	27830	ND	1.17
Black H/S	C	WB06	0.27	6.27	3.00	56.3	4.62	ND	0.79
Yellow Shade Green	C	WB32	12.1	3.60	455	217	12383	ND	178
Orange	C	WB53	0.31	0.44	75	182	4.82	ND	6.77
White	S	151	ND	0.55	8335	25.4	ND	ND	0.90
Super black	S	148	0.33	21.3	6.58	183	1.71	ND	3.45
Blue	S	172	34.2	4.42	9.64	122	56123	ND	ND
Iris blue	S	174	18.7	0.37	0.34	9.59	24653	ND	ND
Emerald	S	177	13.4	4.27	578	455	12089	ND	174
Green toner	S	185	2.08	0.82	46.3	23.2	854	ND	1.94
Orange	S	160	0.28	1.12	0.26	113	1.43	ND	1.03
Brilliant red	S	165	ND	0.30	6.56	21.8	0.16	ND	6.29
Dark yellow	S	159	0.41	0.79	22.4	50.5	0.17	ND	0.42
Light yellow	S	179	1.40	1.60	50.9	53.5	0.31	ND	1.98
Ochre	S	156	11.5	8.85	25.5	14833	0.38	ND	878

[*]Colors and codes are based on manufacturer definition; Samples were obtained from the two service departments of new car dealers (shops W1 and W2), and collected as single-color tinter. Generic binder (50% (w/w)) were added before analysis to mimic actual paints; [**]Brand, C:Cromax, S:Standox; [***] No binder was added. ND:below method detection limit.

Table S3. Worker tasked-based airborne exposures to metal particles during refinish painting

Sample type*	Paint color	Sampling time(min)	Worker**	Air concentration (μg/m^3)						
				Mass (total)	Pb	Cr (total)	Fe	Sr	Cu	Al
**Water-based paint**										
S1	Blue, grey	15	W1-1	1741	ND	ND	ND	ND	ND	ND
S1	Blue, grey	15	W1-1	3202	ND	ND	ND	ND	ND	ND
P1	Black, metallic	16	W1-2	13445	ND	ND	ND	ND	9.98	8.53
P1	Black, metallic	20	W1-2	4253	ND	ND	ND	2.74	1.20	0.80
P2	Black, metallic	18	W1-2	14609	ND	ND	ND	ND	10.31	ND
P1	White	18	W1-3	19872	1.10	ND	69.20	2.75	0.11	73.95
P1	Silver	16	W1-3	12352	ND	ND	27.44	2.06	0.08	309.2
P2	White	19	W1-3	1582	ND	ND	8.26	ND	ND	15.78
S3	Black	15	W2-1	9320	2.03	ND	53.57	ND	ND	66.54
S3	Black	15	W2-2	2329	1.71	0.07	76.43	ND	ND	101.1
S2	Silver	16	W2-3	3432	3.55	0.09	21.55	2.18	2.07	16.85
P1	Black	15	W2-3	5992	0.50	0.86	29.61	ND	ND	ND
P2	Black	15	W2-3	2663	1.53	0.41	0.93	ND	2.44	ND
P1	White, pearl	16	W2-3	9671	1.46	ND	23.10	ND	ND	ND
P2	White, pearl	16	W2-3	12833	2.48	ND	28.33	ND	ND	74.60
P1	Beige+ClearCoat	17	W2-3	6608	1.78	0.05	74.34	ND	ND	130.1
P2	Beige+ClearCoat	18	W2-3	1669	0.77	ND	24.81	ND	ND	27.16
P1	Black	12	W2-3	3537	0.56	ND	9.22	ND	ND	26.56
P2	Black	13	W2-3	1926	1.39	ND	ND	ND	ND	24.41
**Solvent-based paint**										
P1	Black	57	S1-1	4255	0.56	ND	0.47	ND	ND	0.59
P2	Black	57	S1-1	6811	0.54	ND	2.28	ND	ND	0.99
P2	White	66	S1-1	1552	0.54	ND	1.33	ND	ND	9.40
P1	White	18	S1-1	23612	ND	0.31	32.94	ND	ND	104.7
P1	White	16	S1-1	21563	ND	0.91	26.80	ND	ND	105.2
P1	White	16	S1-1	32189	ND	1.49	41.11	ND	ND	139.7
P1	White+ClearCoat	16	S1-1	29532	4.17	1.77	42.55	ND	ND	160.8
S2	Black	9	S2-1	1830	ND	1.36	40.96	ND	ND	2.70
P2	Silver	16	S2-1	471.7	ND	ND	ND	ND	ND	1.01
P1	Silver	14	S2-1	2511	ND	0.16	15.97	ND	ND	208.3
P1	White	19	S2-2	1321	ND	ND	24.65	ND	ND	5.50
P2	White	20	S2-2	10441	ND	ND	21.85	ND	ND	31.32
S1	Mustard	16	S2-3	1569	ND	2.42	28.52	ND	ND	2.83
P1	Black	28	S2-3	16139	ND	0.28	32.11	ND	ND	4.59
P2	Black	28	S2-3	5591	ND	0.09	15.32	ND	ND	2.93
P2	Yellow	64	S5-1	1682	267.2	43.33	1.11	ND	ND	3.23
P1	(Sealant)	10	S5-1	4000	39.06	3.52	20.98	ND	ND	62.56
P1	Yellow,light	13	S5-1	6346	170.2	19.12	15.88	ND	ND	26.89
P1	Yellow	15	S5-1	11167	2345	249.5	11.57	ND	ND	43.56
P1	Yellow	15	S5-1	20000	3593	603.6	8.38	ND	ND	25.88

*S1:scraping old paint, S2:sanding of filling, S3:sanding of new, un-painted bumper, P1:spray painting, personal sampling, P2:spray painting, area sampling inside booth; **Store-individual worker. ND: below method detection limit.
